# Primary Pericardial Mesothelioma: A Rare Entity

**DOI:** 10.1155/2013/283601

**Published:** 2013-06-13

**Authors:** Mohit Godar, Jianhua Liu, Pengguo Zhang, Yang Xia, Qinghai Yuan

**Affiliations:** ^1^Department of Radiology, Norman Bethune College of Medicine, The Second Hospital of Jilin University, 218 Ziqiang Street, Nanguan District, Changchun, Jilin 130041, China; ^2^Department of Pathology, Norman Bethune College of Medicine, The Second Hospital of Jilin University, 218 Ziqiang Street, Nanguan District, Changchun, Jilin 130041, China

## Abstract

Primary pericardial malignant mesothelioma is an extremely rare neoplasm that arises from the pericardial mesothelial cell layers. Clinical symptoms and signs are frequently nonspecific, and the diagnosis is usually made after surgery or at autopsy. There is no standard treatment for pericardial mesothelioma; nonetheless, radical surgery is the mainstay of therapy for localized disease. The neoplasm is highly aggressive and carries a dismal prognosis with an overall survival of less than six months. This paper presents a case study of a 68-year-old patient with a primary pericardial malignant mesothelioma. Radiologic evaluation revealed a small nodule in the posterior pericardium with pericardial and bilateral pleural effusions. The diagnosis was established after surgery by histological and immunohistochemical studies. The patient remained alive and free of disease for about 24 months; however, due to rapid local recurrence, the patient died 27 months after the surgical treatment.

## 1. Introduction

Primary cardiac tumors of heart are uncommon, whereas primary pericardial mesothelioma (PPM) is exceptionally rare with an incidence of only 0.0022% in a series of nearly 500,000 random autopsies [1]. PPM accounts for 2-3% of the primary heart and pericardial tumors; it is the third tumor after angiosarcoma (33%) and rhabdomyosarcoma (20%) [2]. The diagnosis of PPM is usually difficult and late, and in the majority of cases, the diagnosis of PPM is typically made at autopsy [3]. The optimal treatment for PPM has not been well established. Owing to poor response to existing systemic and radiation therapy, radical surgery remains the cornerstone of treatment in an attempt to cure the localized disease. The neoplasm is very aggressive, and the prognosis has been uniformly dismal for the only handful of reported cases surviving more than 1 year after diagnosis. We herein describe a 68-year-old man who underwent surgery for localized pericardial mass found on radiologic assessment, and subsequently diagnosed as PPM. Twenty-seven months after surgery, the patient died due to local recurrence. 

## 2. Case Presentation

**Figure 1:**
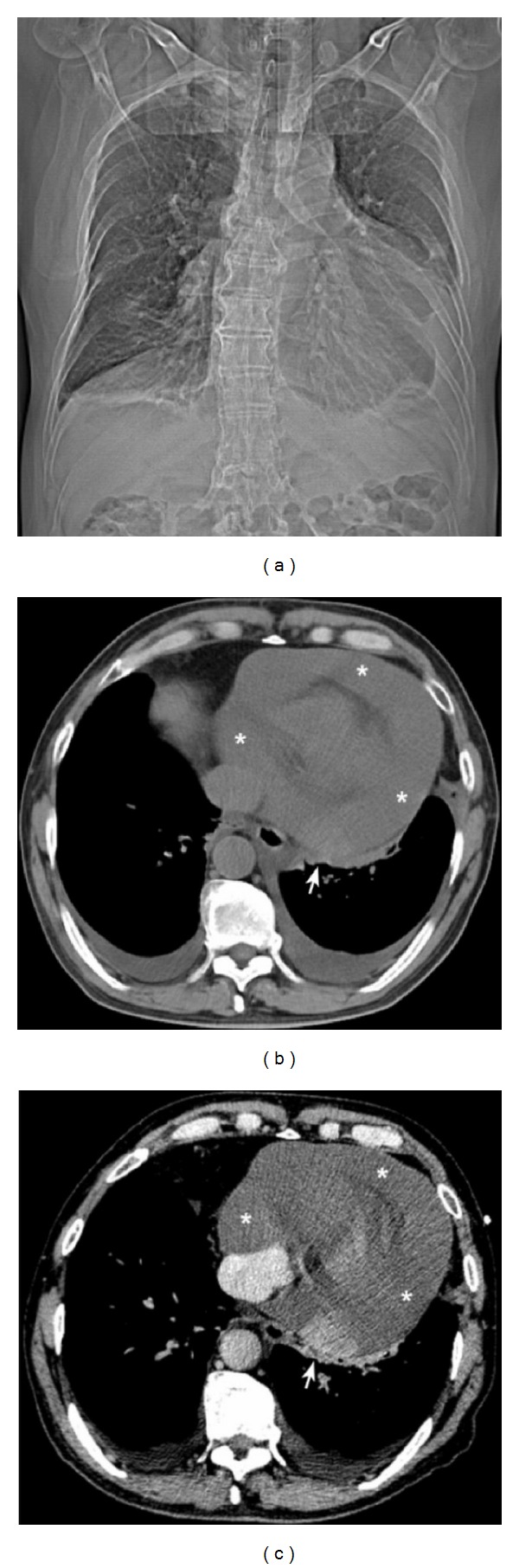
(a) Computed tomography (CT) scanogram/scout shows enlarged cardiac silhouette with blunting of the left costophrenic sulcus. (b) Nonenhanced chest CT image reveals a nearly isoattenuating nodular mass arising from the left posterior pericardial wall (arrow) with a large circumferential pericardial effusion (asterisks) and bilateral pleural effusions without signs of cardiac tamponade. (c) Contrast-enhanced chest CT scan shows a well-defined, enhancing nodule (arrow).

A previously healthy 68-year-old man came to our hospital in May 2010 with a 1-month history of malaise, dyspnea on exertion, bilateral lower-extremity edema, and a 5-kilogram weight loss. His past medical history was unremarkable. The patient was a nonsmoker and denied history of prior asbestos exposure. The physical examination was notable for bilateral pitting edema of lower legs and muffled heart sounds on cardiac auscultation. The hematologic and biochemical studies were unremarkable. Electrocardiography was nonspecific except for low-voltage QRS complexes, and a chest radiograph revealed an enlarged cardiac silhouette. Transthoracic echocardiography revealed a circumferential pericardial effusion with a heterogeneous hyperechoic mass in the posterior pericardial wall without signs of cardiac tamponade. Computed tomography (CT) scan of the chest and abdomen was performed to determine the extent of the disease which demonstrated a nearly isoattenuating nodular mass, around 3.6 cm × 2.2 cm in diameter, arising from left posterior pericardial wall and a pericardial effusion encasing the heart with small bilateral pleural effusions. The lesion showed enhancement on contrast-enhanced CT image (Figure 1). Aside from the above-mentioned findings, no other abnormalities were detected on CT examination of the chest and abdomen. 

**Figure 2:**
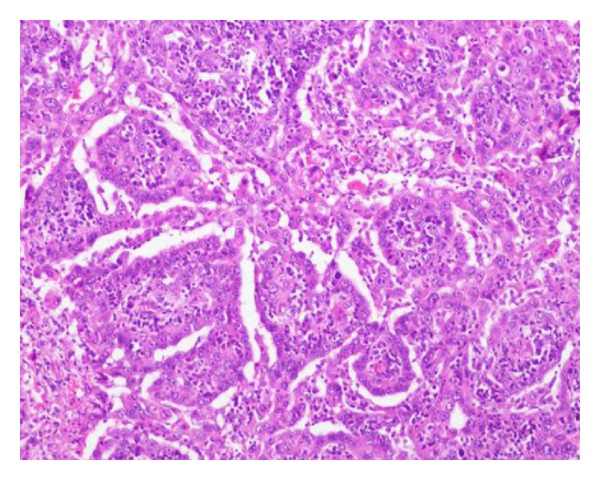
Histopathologic examination of postpericardiectomy specimen reveals abundant pleomorphic malignant cells with epithelial appearance (hematoxylin and eosin, ×100).

**Figure 3:**
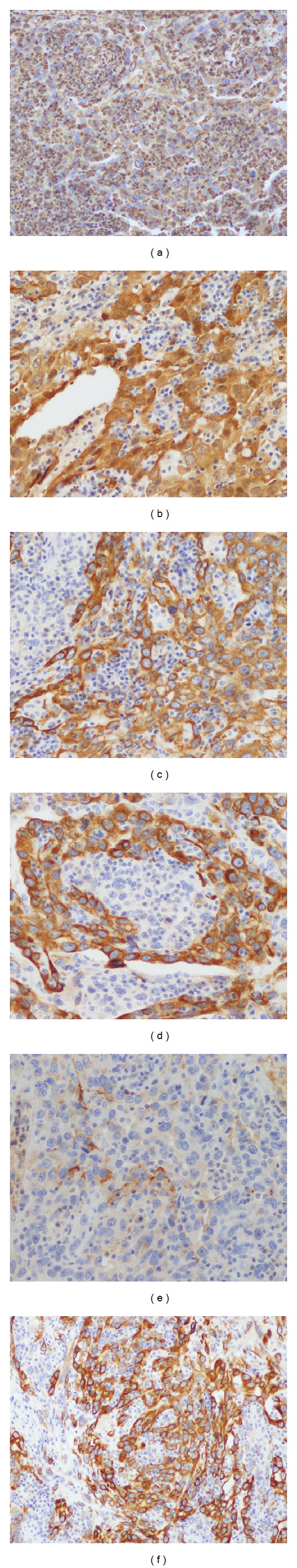
The tumor cells are strongly and diffusely positive for vimentin (a), calretinin (b), CK AE1/AE3 (c), and CK7 (d), with focal patchy immunoreactivity for HBME-1 (e) and CK 5/6 (f). (×200).

**Figure 4:**
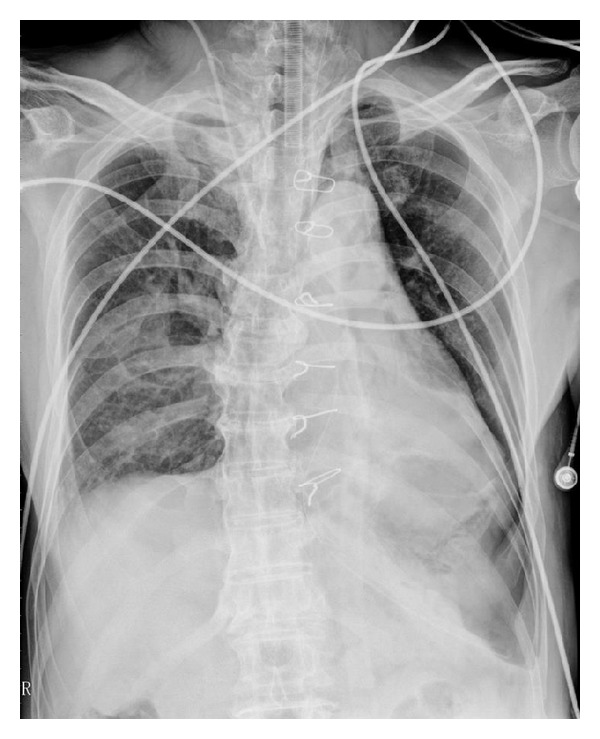
Anteroposterior chest radiograph on the first postoperative day shows a decrease in cardiac silhouette with increased lung markings.

Primary pericardial malignancy was suspected based on the imaging results; consequently, the patient underwent surgery. Grossly, the tumor was 3.5 cm in diameter, well encapsulated with a significant serosanguinous pericardial effusion which was drained. The cut surface of the tumor was fleshy and grey-white to yellowish in color without any signs of invasion or infiltration to adjacent organs. Cytological and microbiological analyses of pericardial fluid were noncontributory. Pathological evaluation of the postpericardiotomy specimen confirmed the diagnosis of pericardial mesothelioma, epitheloid type. The epitheloid cells had eosinophilic cytoplasm and open nuclei with prominent nucleoli, exhibiting tubulopapillary patterns of growth with connective tissue cores accompanied by a lymphoplasmacytic infiltration. Mitoses were frequent (Figure 2). On immunohistochemistry, neoplastic cells were strongly and diffusely positive for vimentin, calretinin, CK AE1/AE3, and CK7, with focal patchy immunoreactivity for HBME-1 and CK 5/6 (Figure 3), and negative for TTF-1, PLAP, CEA, CD5, CD30, and CD 117. Postoperative recovery was uneventful (Figure 4), and subsequently, our patient received two cycles of chemotherapy of cisplatin and pemetrexed without any complications. The patient remained alive and free of disease for around 24 months; however, in August 2012, he died due to rapid local recurrence, 27 months after surgery. 

## 3. Discussion 

Malignant mesothelioma is an uncommon neoplasm that derives from the mesothelial cells linings of pleural, peritoneal, or pericardial surfaces. About 65%–70% of malignant mesotheliomas arise from pleura and 30% from peritoneum, whereas mesothelioma originating from the pericardium or tunica vaginalis testis is rare and accounts for 1%-2% of all mesotheliomas [4]. Pericardial masses are usually metastatically spread originating from lung or breast cancer, melanoma, lymphoma, or acute leukemia [5]. Primary tumor such as pericardial mesothelioma is a rare entity and occurs over a wide age range (3–80 years) with a median age of 46 and is more common in men than women (2 : 1) [6]. The precise etiology of PPM remains uncertain. Unlike pleural and peritoneal mesothelioma, the relationship between asbestosis exposure and PPM is controversial. A literature review of 29 cases from 1993 to 2008 revealed that only 3 out of 14 cases with known exposure to asbestosis were associated with PPM [6]. In the present case, the patient has categorically denied prior asbestosis exposure. Other suspected risk factors are radiation exposure, SV40 virus, TB, and exposure to nonasbestos mineral fiber such as erionite [7]. 

Typically, the onset of PPM is insidious in nature with nonspecific symptoms such as cough, dyspnea, orthopnea, chest pain, and sometimes paradoxical pulse [8], that can be accompanied by constitutional symptoms such as fever, night sweats, weight loss, and generalized weakness. These symptoms and signs are most often attributed to compression or constriction of the heart caused by effusions or tumor invasion/infiltration to adjacent structures. Common clinical presentations of pericardial mesothelioma are constrictive pericarditis, pericardial effusion, cardiac tamponade, and congestive heart failure [3, 8]. Metastases are present in around 25–45% of the cases involving regional lymph nodes, lungs, and kidneys [2].

The diagnosis of pericardial mesothelioma is usually difficult and late, and to date, about 200 cases of PPM have been reported in the medical literature with only 25% of these being diagnosed antemortem [3, 9]. Pericardial mesothelioma presents with focal or diffused pericardial thickening and a variable pericardial effusion. Chest radiograph typically shows enlarged cardiac silhouette, evidence of pericardial effusion, or diffuse mediastinal widening. Echocardiography is the most commonly used initial cardiac imaging modality. However, computed tomography and magnetic resonance imaging are generally required to provide additional information about size, location, and extent of pericardial involvement. Further, echocardiography and CT are useful in guiding pericardiocentesis. Although it is difficult to aspirate, cytological examination and high pericardial hyaluronic acid content of the pericardial aspirate can be diagnostic of PPM [3]. However, the diagnostic yield of pericardial fluid cytology is often poor with only 24% of the cases presenting malignant cells in a literature review of 17 patients with PPM [6]. Therefore, in most cases of PPM, the definitive histological diagnosis can be obtained either after surgery or at autopsy. PPM can cause focal increased uptake of radiotracer on positron emission tomography/computed tomography scan (PET/CT) [9], which can be used in distinguishing whether a nodule or mass is benign or malignant. Histological and immunohistochemical studies of pericardial mesothelioma are similar to that of pleural mesothelioma. The tumor cells may have three distinct patterns, that is, predominantly epithelial, predominantly fibrous (spindle cell), and biphasic (mixed) [6]. Negative adenocarcinoma markers, such as carcinoembryonic antigen (CEA) and positive mesothelial markers, for example, calretinin, and cytokeratins 5/6 are useful in differentiating mesotheliomas from adenocarcinomas [10]. 

Owning to rarity, there is no standard treatment regimes for PPM. Surgical resection remains the main treatment modality in PPM, and may be curative for the localized disease [3, 6, 8, 9]. Nonetheless, in most cases, it is not possible to remove tumor completely, and therefore, PPMs generally are treated with a palliative intent based on surgery, chemotherapy, and radiotherapy. The overall prognosis of PPM remains dismal owing to its late presentation, inability of complete tumor eradication by surgery, and the poor response of tumor cells to radiotherapy or chemotherapy, with median survival times of six months from diagnosis [2, 3, 5, 6, 8, 9]. The most common causes of death are cardiac tamponade, vena cava occlusion, and congestive heart failure [2].
